# Ancient Immunity. Phylogenetic Emergence of Recognition-Defense Mechanisms

**DOI:** 10.3390/biology10040342

**Published:** 2021-04-19

**Authors:** Loriano Ballarin, Matteo Cammarata, Pierangelo Luporini

**Affiliations:** 1 Department of Biology, University of Padova, 35131 Padova, Italy; 2 Department of Earth and Sea Sciences, University of Palermo, 90128 Palermo, Italy; matteo.cammarata@unipa.it; 3 Department of Biosciences, University of Camerino, 62032 Camerino, Italy; piero.luporini@unicam.it

Although still scarcely considered by the majority of the biomedical world, invertebrates have greatly contributed to the elucidation of fundamental biological problems. Let us think to the birth of cellular immunology with Mečnikov’s experiment on sea star larvae, or the importance of squids for the comprehension of the molecular basis of nerve conduction, or the contribution of sea urchins to the studies of fertilization and early development. Indeed, invertebrates represent the overwhelming majority of animal biodiversity and this simple assumption can explain the growing interest of the scientific community towards various aspects of their biology. Invertebrate immunobiology is one of such aspects.

Invertebrate immunity is of the innate type, which means without the somatic recombination events occurring in lymphocytes of jawed vertebrates and accounting for the high specificity of their responses. However, even if lacking the adaptive branch, this does not mean that invertebrate immune systems are as simple as originally thought. Indeed, the huge variety of adaptations led to a high diversification of the responses, and this Special Issue, including two research papers and six reviews, presents some examples.

In their research paper [1], Peronato et al. report on the identification of a receptor for C3a/C5a in the colonial ascidian *Botryllus schlosseri*, a reliable model for immunobiology studies. Named BsC3a/C5aR, it is a new member of the G-protein-coupled receptor family. The gene for BsC3a/C5aR is actively transcribed in the cytotoxic cells known as morula cells, deeply involved in inflammatory reactions such as those occurring during the allorejection between contacting, genetically incompatible colonies. Its transcription is significantly reduced during the takeover phase (generation change) of the colonial blastogenetic cycle, when *bsc3* (the gene for the complement factor C3 of *Botryllus*) is over-transcribed, suggesting the presence of a negative autocrine feedback loop blocking the transcription of *bsc3a/c5ar* in the presence of a high amount of BsC3 in the circulation, so to prevent a diffuse inflammation. The presence of regulatory loops influencing the transcription of *bsc3* and *bsc3a/c5ar* is further supported by the observation that the C3aR agonist can enhance the transcription of *bsc3*.

The second research paper, by Montanari et al. [2], reports new findings on immune responses in the gastropod mollusk *Pomacea canaliculata* towards a nematode-based molluscicide, used as method of control of crop pests: it can induce the transcription of an orthologue of the mammalian bactericidal/permeability increasing protein (BPI) gene. As expected, the hemocytes are the cells involved in immune responses to the nematode worms, and their presence is particularly high in organs such as the anterior kidney and the gills.

In her review [3], Nydam gives a detailed account of the distribution of coloniality and allorecognition (in the form of colony specificity) in the various clades of ascidians. In colonial ascidians, allorecognition is a complex phenomenon, controlled by specific genes (the formal genetics of fusibility is known only in the compound ascidian *Botryllus schlosseri*) leading to the fusion of contacting colonies in a single chimeric colony, or their rejection and the consequent triggering of an inflammatory reaction. The latter is mediated by the cytotoxic morula cells that degranulate and release the enzyme phenoloxidase, with the consequent induction of oxidative stress along the contact border. As a second step in allorecognition, the progressive resorption of one of the fused partners in the chimeric colony frequently occurs. Allorecognition probably appeared as a tool to regulate gamete compatibility and self-fertilization, as suggested in this paper, or for the protection against predatory cell lineages of the same species in chimeras.

The review by Auguste et al. [4] presents various kinds of cell communication systems in invertebrate defense, such as the formation of extracellular traps (ETs), exosomes and tunneling nanotubes (TNTs). ETs are web-like structures composed of chromatin, heavily impregnated with antimicrobial proteins, able to capture, neutralize and kill a variety of pathogens. They immobilize microbes, preventing their dissemination, and expose them to a high, localized concentration of antimicrobial proteins. Up to now, they have been poorly described in invertebrates, although recent reports indicate that ETs play a crucial role in the defense mechanisms also in these animals. Exosomes are secreted membrane vesicles, 30–150 nm in size, generated through an endosomal pathway mediated by multivesicular bodies. In mammals, exosomes participate in responses to viral and bacterial infection, take part in antigen presentation and stimulate the release of inflammatory factors and the expression of immune molecules. In addition, exosomes of infected cells can deliver pathogen-associated molecular patterns (PAMPs) and host–derived pattern recognition receptors (PRRs) leading to the activation of innate immunity and inflammatory responses. Again, little investigation has been devoted to the study of exosomes in the invertebrate immune response. However, recent literature data, mainly referring to cnidarians, nematodes, annelids, bivalves, crustaceans and insects, are partially retrieving this lack of information. TNTs are thin membranous structures that allow the transfer of signals, from ions to vesicles and organelles, by direct cell contact. They have been described in various mammalian cells types and in almost all immune cells, both under physiological and stress conditions. Recently, they have been described also in insects and bivalve mollusks. Future investigations on invertebrate TNTs can unravel unexpected aspects of the complexity of their immune responses.

The next review [5] provides an exhaustive description of cnidarian immunity, with particular focus on anthozoans. Cnidarians are non-bilaterian animals lacking an internal circulatory system and their immunity relies mainly on epithelial cells. They use a variety of receptors (e.g., Toll-like receptors, lectins, complement factors and scavenger receptors) to cope with the non-self and assure the integrity of the endosymbionts that, in anthozoans, are important for survival. The authors start with a wide survey of the main phenomena in which pattern recognition receptors (PRRs) are involved, such as allorecognition and symbiotic interactions with algae and the microbiota. The maintenance of symbiosis is associated with the downregulation of host immunity, as suggested by the observation that bleached animals are less susceptible to infection by pathogens. This supports the idea of a tradeoff between zooxanthellae (and other symbiotic organisms) and immune functions. Then, they present the repertoire of known PRRs and signaling pathways of anthozoans, many of which show homologies and identities with their mammalian counterparts. The effector responses are the subjects of the last part of the review, including humoral/mucosal and cellular immune responses, with a particular focus on inflammation. Here, data concentrate on the effects of the inoculation of various substances in *Anemonia viridis*, leading, in most cases, to the appearance of a rejection zone in the pedal disc, a defense mechanism never described before in cnidarians.

In their review [6], Bodó et al. report on the effects of silver and carbon nanomaterials on annelid immunity, with particular reference to antioxidant responses. The authors concentrate their attention on terrestrial and freshwater annelids (earthworms and leeches) considered “keystone” species to evaluate the health of ecosystems. In both these groups, nanomaterials have similar detrimental effects, inducing inflammatory responses and an increased production of reactive oxygen species. Nanomaterials are known to induce the formation of protein corona, likely involved in the uptake of nanomaterials by endocytosis. The induction of protein corona has been fully ascertained in earthworms, where researchers are trying to identify them and define their biological role. Conversely, nothing is known on protein corona formation and nanoparticle uptake mechanisms in leeches.

The following review [7] aims to present the current knowledge on the role and modulation of allograft inflammatory factor-1 (AIF-1) in invertebrates as compared to vertebrates. AIF-1 is a calcium-binding scaffold/adaptor protein, frequently associated with inflammatory diseases and extensively studied in vertebrates, especially in mammals, where it has an immunomodulatory role and is expressed by activated macrophages, microglial cells and dendritic cells. Its amino acid sequence is well conserved in vertebrates and invertebrates: this suggests its involvement also in invertebrate inflammatory responses. Indeed, among invertebrates, AIF-1 is known to be involved in the innate immunity of sponges, cnidarians, annelids, mollusks and echinoderms, and, in many cases, it is upregulated in response to biotic and physical challenges. AIF-1 activity is clearly linked to inflammation and immune response events in all the animal phyla in which the protein has been characterized. However, its ubiquitous expression in various invertebrate tissues suggests its participation in a variety of other processes, where its role remains largely unknown and deserves further studies.

The aim of the last review [8] is to trace the evolutionary history of the immunoglobulin molecule. By considering a wide range of prokaryotic and eukaryotic organisms and operating a wide and detailed survey of the literature of interest, the authors consider the antibody molecule architecture as a modular and dynamic structure requiring various components (Ig fold, IgV domain, somatic recombination and hypermutation) as structural elements of the immunoglobulins, many of which presented in living organisms well before the appearance of jawed vertebrates. The depicted scenario is of great interest from an evolutionary point of view and paves the way to additional future insights.

We hope that this Special Issue, stemmed from the activity of the Italian Association of Developmental and Comparative Immunobiology, can find the interests of a large audience, and pave the way for future cultural exchanges among invertebrate immunologists and fruitful collaborations between them and colleagues of the medical schools, towards a better comprehension, at various levels of investigation, of the disparate strategies set up by organisms to cope with the non-self.

## References

[1] Peronato A., Franchi N., Ballarin L. (2020). Insights into the complement system of tunicates: C3a/C5aR of the colonial ascidian *Botryllus schlosseri*. Biology.

[2] Montanari A., Bergamini G., Ferrari A., Ferri A., Nasi M., Simonini R., Malagoli D. (2020). The immune response of the invasive golden apple snail to a nematode-based molluscicide involves different organs. Biology.

[3] Nydam M.L. (2020). Evolution of Allorecognition in the Tunicata. Biology.

[4] Auguste M., Balbi T., Ciacci C., Canesi L. (2020). Conservation of cell communication systems in invertebrate host–defence mechanisms: Possible role in immunity and disease. Biology.

[5] Parisi M.G., Parrinello D., Stabili L., Cammarata M. (2020). Cnidarian immunity and the repertoire of defense mechanisms in anthozoans. Biology.

[6] Bodó K., Baranzini N., Girardello R., Kokhanyuk B., Németh P., Hayashi Y., Grimaldi A., Engelmann P. (2020). Nanomaterials and annelid immunity: A comparative survey to reveal the common stress and defense responses of two sentinel species to nanomaterials in the environment. Biology.

[7] Vizioli J., Verri T., Pagliara P. (2020). Allograft inflammatory factor-1 in metazoans: Focus on invertebrates. Biology.

[8] Oreste U., Ametrano A., Coscia M.R. (2021). On origin and evolution of the antibody molecule. Biology.

